# Effects of Foods of Mesoamerican Origin in Adipose Tissue and Liver-Related Metabolism

**DOI:** 10.3390/medicina59111907

**Published:** 2023-10-28

**Authors:** Alejandra Meza-Rios, Erika Fabiola López-Villalobos, Luis Alberto Anguiano-Sevilla, Sandra Luz Ruiz-Quezada, Gilberto Velazquez-Juarez, Rocío Ivette López-Roa, Ana Laura Marin-Molina, Adelaida Sara Minia Zepeda-Morales

**Affiliations:** 1 Laboratorio de Análisis Clínicos y Bacteriológicos (Vinculación), Departamento de Farmacobiología, Centro Universitario de Ciencias Exactas e Ingenierías (CUCEI), Universidad de Guadalajara, Boulevard Marcelino García Barragán, No. 1421, Guadalajara 44430, Mexico; alejandra.mezarios@academicos.udg.mx (A.M.-R.); erika.f_lopez@hotmail.com (E.F.L.-V.); analaura_marin@hotmail.com (A.L.M.-M.); 2 Departamento de Farmacobiología, CUCEI, Universidad de Guadalajara, Blvd. M. García Barragán, No. 1421, Guadalajara 44430, Mexico; alberto.anguiano@academicos.udg.mx (L.A.A.-S.); sandra.ruiz@academicos.udg.mx (S.L.R.-Q.); 3 Laboratorio de Análisis Fisicoquímicos Externos, Departamento de Química, CUCEI, Universidad de Guadalajara, Blvd. M. García Barragán, No. 1421, Guadalajara 44430, Mexico; gilberto.velazquez@academicos.udg.mx; 4 Laboratorio de Investigación y Desarrollo Farmacéutico, Departamento de Farmacobiología, CUCEI, Universidad de Guadalajara, Blvd. M. García Barragán, No. 1421, Guadalajara 44430, Mexico; rocio.lopez@academicos.udg.mx

**Keywords:** Mesoamerican foods, metabolism, liver disease, adipose tissue, obesity, milpa

## Abstract

Adipose tissue and liver metabolism play a key role in maintaining body homeostasis; therefore, their impairment conduces a pathological state. Nowadays, occidental lifestyle is a common etiological issue among a variety of chronic diseases, while diet is a unique strategy to prevent obesity and liver metabolism impairment and is a powerful player in the treatment of metabolic-related diseases. Mesoamerican foods are rich in bioactive molecules that enhance and improve adipose tissue and liver performance and represent a prophylactic and therapeutic alternative for disorders related to the loss of homeostasis in the metabolism of these two important tissues.

## 1. Introduction

The liver is the main organ responsible for biochemical metabolism in the human body and it participates in energy metabolism and maintains metabolism homeostasis. The liver metabolizes either beneficial or harmful compounds and endogenous and exogenous molecules. Compounds absorbed by the intestine including drugs and nutrients, which first pass through the liver to be processed into smaller products, thereby sustaining and controlling their concentrations in the bloodstream [1,2]. Biomolecules such as lipids and carbohydrates components form the diet are metabolized by the liver to generate the necessary energy, which affects several physiological processes participating in body homeostasis. Therefore, a dysfunction in hepatic metabolism can generate several hepatic diseases including (a) metabolic-associated fatty liver diseases (MAFLD), (b) alcoholic liver disease, (c) fibrosis and cirrhosis, and (d) hepatocellular carcinoma (HCC) [2]. MAFLD nowadays is the principal chronic liver disease [3,4]. MAFLD represents a spectrum of diseases characterized by an excessive accumulation of rich lipid droplets in the hepatocytes, called hepatic steatosis, as a result of their deficient synthesis or utilization [4,5], which is a process considered part of the metabolic syndrome. In addition, non-alcoholic steatohepatitis (NASH) is the pathogenesis evolution in MAFLD patients that may evolve to fibrosis, cirrhosis, and hepatocellular carcinoma [4].

On the other hand, obesity is defined by the WHO as the excessive accumulation of fat that can impair health. The fundamental cause of obesity is an energy imbalance between calories consumed and calories expended. Obesity has been associated with a decreased life expectancy by 5 to 20 years [6]. In the past 50 years, the prevalence of obesity has increased across the world to pandemic status, with the worldwide increased rate prevalent among children and adolescents [6]. Nowadays, we are living in obesogenic environments that have effects on our behavior and lifestyle, including a reduction in home cooking, reduced physical activity, increased habit of consuming non-healthy snacks and sweets, soft drinks, and fast-food consumption [6], where the increased intake of foods high in fat and sugar is the principal contributor of obesity [7].

It has been established an association between dietary intake habits and the development of disease features in patients with MAFLD. Western countries consume a diet where almost 40% of the energy comes from fat and 0.2% from cholesterol [5]. Therefore, dietary changes, lifestyle modification, and weight loss are the first-line therapies for MAFLD/NASH [6,8,9]. Western-type diets have been developed to mimic the human diet in animal research, achieving some features of MAFLD in wildtype rodents [5].

Studies on food and metabolic profiles showed that, apart from macro and micro-biomolecules like proteins, lipids, carbohydrates, minerals and vitamins, wild and natural foods have bioactive molecules that modulate the metabolic profile or/and protect against pathological conditions, particularly non-communicable diseases. These bioactive components activate or inhibit gene expression of molecules that change the metabolic response to macronutrients and generate the feeling of satiety [10]. Currently, the Mediterranean diet has been recognized and adopted as an intervention diet for the management of MADLF, obesity, and other pathologic conditions [9,11,12,13]. Nevertheless, different regions might have their own “healthy” food products based on their climate and culturally and traditions [11], like Mesoamerican foods, including the milpa agroecosystem. This review focuses on some of the Mesoamerican foods that have been shown to have beneficial effects on adipose tissue and liver metabolism features.

## 2. Mesoamerican Food and Mexican Milpa Agroecosystem

Some international health organizations are promoting traditional diets to fight the growth in non-communicable diseases and obesity rates around the world [11,14]. Traditional diets generally are healthy diets because their core are plant-based foods like grains, legumes, vegetables, fruits, and tubers, with low consumption of foods from animal origins [11,14,15,16], like Mesoamerican diet. Culturally Mesoamerica includes the present day middle and south Mexico, Belize, Guatemala, parts of Honduras, and El Salvador; geographically Nicaragua, Costa Rica, and Panama are also included in the region [17].

**Figure 1:**
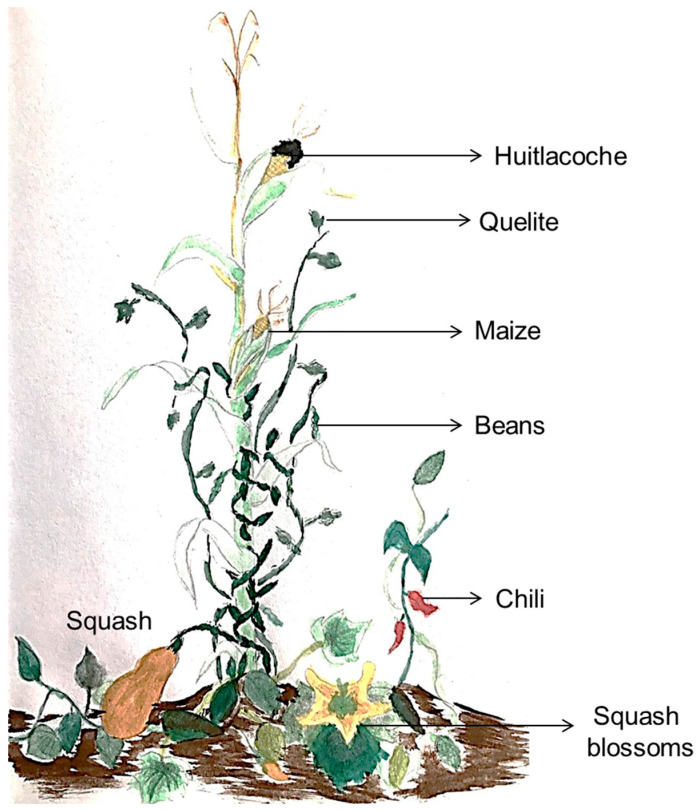
Milpa illustration. The image represents the main components of the milpa. Maize, beans, squash and chili, and some additions like squash blossoms, quelites, and huitlacoche; all ingredients of the traditional Mexican cuisine.

On the other hand, the milpa is an ancestral and millenary maize-based agroecosystem in Mexico and Mesoamerica characterized for been a polyculture system composed mainly by an intercropping of maize, beans, squash, and chili, and some other additions like tomato, jicama, squash blossoms, “quelites”, “huitlacoche” and avocado, among others (Figure 1). The milpa has been recognized as an invaluable repository of cultural and biological diversity [18,19]. The implementation of this agroecosystem can guarantee food and economic security and preserve non-economic cultural values for the communities that used such a system [19]. On this basis, the milpa practice is the foundation of food security in many Latin American rural regions [20].

In the effort to define the traditional Mexican diet, Valerino-Perea and colleagues [11] conducted a systemic review across 61 scientific documents and found that the representative food groups and their components are the following: (a) grains and tuber, like maize, amaranth, rice, wheat, yucca, potato, and sweet potato; (b) maize products, like tortilla, tamales, and atole; (c) legumes, mainly beans; (d) vegetables such as squash, chayote, nopales, maguey, tomato, tomatillo, quelites; (e) fruits composed by anona, capulín, guava, jicama, mamey, plums, prickly pear, zapote; (f) meats from turkey, chicken, ducks, venison, dog, rabbit, hare, armadillo, and beef; (g) herbs and condiments like chili, epazote, vanilla, salt, and onion; (h) oils and fats from avocado, pumpkin seed, and chia seeds; (i) beverages of chocolate drinks and pulque (fermented maguey drink); (j) insects, for example grasshoppers and locusts, maguey worms, ants and their larvae, chicatanas, and escamoles; and (k) sweets and sweeteners like honey, sugar, and sugarcane. It is important to note that some of the foods cited are not from Mexican origin; they were adopted from other countries and even from other continents. In addition, the authors presented findings on the association of the traditional Mexican diet and health issues where some studies reported differences in diabetes-related outcomes, others reported differences in obesity, and one study presented differences in dyslipidemia [11]. Also, Mexican epidemiological studies showed that people which include traditional foods in their diet have a lower risk of developing type 2 diabetes mellitus [10].

Crocker and colleagues studied an indigenous community in Mexico where they observed that some of their population changed the habit to eat “nixtamalizado” maize, an ancient technique to prepare maize with lime and a process that conserved all the maize nutrients and the properties of the whole grain cereal, and instead used industrialized maize flour with a low content of fiber, essential oils, and calcium. Apart from the maize, they abandoned the consumption of Mesoamerican vegetables like nopales, quelites, mushrooms among others, and have been increased the consumption of refine foods like sodas; a change that has impacted the nutritional status of the people of this region, where 24% of the women who had adopted a Western diet had obesity combined with a short stature [21].

## 3. Scientific Evidence of Metabolic Liver Improvement by Mesoamerican Origin Foods and Their Effects in Adipose Tissue and Liver Pathogenic State

### 3.1. Cacao Bean

The cacao bean (*Theobroma cacao*) is a fruit with the highest amount of flavanols of all foods per weight basis. The Criollo cacao bean is native to Mexico, Central and South America, and along with maize, is an ancient cultivar. More than 200 molecules have been described in cacao beans; the polyphenol content is about 12–18% of the dry weight, where 60% of the total polyphenols corresponds to monomeric and oligomeric flavanols. The principal monomeric flavanols are (−)-epicatechin, (+)-catechin, and procyanidin B2 (Table 1) [22,23]. Flavanols act as natural antioxidants and interact with signaling proteins, enzymes, DNA, and membranes [22,24]. Therefore, most of the direct effects of cacao flavanols are related to their antioxidant capacity acting as electron donors stabilizing free radicals [22]. In addition, cacao polyphenols activate the redox-sensitive transcription factor nuclear factor E2 related factor 2 (Nrt2) inducing the transcription of antioxidant enzymes including superoxide dismutase, glutathione peroxidase, and heme oxygenase 1. These molecules block the production of nitric oxide synthase (NOS) and reactive oxygen species (ROS), decreasing oxidative stress [22,25].

Gu et al. [26] evaluated the effect of 8% cocoa powder supplementation on obesity-related inflammation in high-fat-fed obese mice. Based on their results, they concluded that cocoa ameliorates inflammation, insulin resistance, and fatty liver disease, all related with obesity, mainly via downregulation of pro-inflammatory genes. In addition, Jang et al. [27] reported that theobromine, a component in cocoa bean, reduced the expression of key adipogenic transcription factors, and regulated lipolysis and fat oxidation in vitro, suggesting that cocoa could improve systemic lipid metabolism protecting against obesity and other metabolic disorders. Also, human consumption of dark chocolate (derived from cacao beans) can reduce oxidative stress lowering the activation of NOX2 in patients with non-alcoholic fatty liver disease, a common hepatic disease related to obesity [28].

Coronado-Cáceres et al. evaluated the properties of cocoa proteins to reduce factors related to obesity and turn on related genes targets against white adipose tissue (WAT) dysfunction in a murine obesity model [29]. Among the results, the supplementation with cocoa proteins prevent body weight gain, reduces serum triglycerides, insulin, leptin, and non-esterified fatty acid levels, and pro-inflammatory molecules; on the other hand, cocoa proteins increase HDL levels, the supplementation upregulated PPARƔ, PPARα, AMPK, Plin1, SIRT1 and PGC-1α, and downregulated TNF-α, Leptin, ACC, and SREBP-1c, molecules related to WAT dysfunction related to obesity. Cocoa proteins downregulated factors related to lipogenesis and upregulated molecules related to energy expenditure, reducing the systemic release of triglycerides and non-esterified fatty acid, and decreasing the pro-inflammatory response [29]. The same group of researchers from Coronado-Cáceres published in 2021 results of their research focused on the effect of cocoa proteins on blood pressure. The result of the effect of the protein fraction of cocoa bean extract on the blood pressure parameter was evaluated by different technologies including in silico, in vitro, and in vivo models. The results showed that the consumption of cocoa proteins blocked the angiotensin-converting enzyme and consequently lowered blood pressure. The authors suggest that the equivalent dose in humans of these proteins in relation to those tested in vivo (rat model) is 1.45 g per day for adults weighing 60 kg [30].

### 3.2. Nopal

Nopal (*Opuntia ficus indica*) is a member of Cactaceae family, and a vegetable extensively consumed in Mexico. It is native to American continent. This vegetable has been recognized as a functional food (a food that benefits human health in addition to the effect of their nutrients alone) based on its high content of fiber, antioxidant molecules like vitamin C, and other bioactive molecules including polyphenols/flavonoids (Table 1) [23].

Rosas et al. evaluated the effect of supplementation of a mixture of Nopal, Cacao bean, and cricket powder (MexMix) in lipid and obesogenic pathways in mice fed with a high-fat/high-sugar diet [23]. Among the results, MexMix showed significantly reduced body weight, liver weight, and visceral/epididymal fat versus high-fat/high-sugar mice. Also, levels of serum lipids such as triglycerides, cholesterol, and LDL cholesterol, insulin, glucose, leptin, resistin, among others, were significantly reduced. On the other hand, MexMix showed an effect on gut microbiota, increasing bacteria involved in beneficial metabolic effects like *Lachnospira*, *Eubacterium coprostanoligenes*, and *Blautia* [23].

Morán-Ramos et al. evaluated the effect of nopal intake on the development of liver oxidative stress and hepatic steatosis, and on the expression of regulation of genes related in hepatic lipid metabolism. The author used a model of obese Zucker rats fed with a control diet or a diet containing 4% nopal. Among the results, the author described a reduction of 50% in hepatic triglycerides levels, lower levels of biomarkers of hepatocyte injury (ALT, AST), reactive oxygen species, hepatomegaly, lipid peroxidation molecules, less postprandial serum insulin levels, and an attenuation of liver steatosis by nopal consumption. Also, nopal administration increased serum levels of adiponectin, and mRNA of genes involved in lipid oxidation and exportation. Based on the results, the author suggests that nopal consumption decreases hepatic steatosis by increasing fatty acid oxidation, lowering oxidative stress, and enhancing liver insulin signaling in obese Zucker rats [31].

Sánchez-Tapia and colleagues investigated if nopal administration could reduce the metabolic consequences of obesity by modifying the intestinal microbiota and preventing metabolic endotoxemia in an animal model of rats fed with a high-fat/high-sucrose diet. The rats were fed with a high-fat/high-sucrose diet for 7 months; after this period, the authors included 5% nopal for 1 month in the diet of the treatment group concomitant with the high-fat/high-sucrose diet. The results showed that nopal modified the gut microbiota composition and increased intestinal occludin-1, reducing the intestinal leaking and metabolic endotoxemia. Also, nopal increased glucose insulinotropic peptide, glucose intolerance, lipogenesis, and metabolic inflexibility. Nopal supplementation reduced oxidative stress in adipose tissue and brain, and hepatic steatosis, improved cognitive response [7].

### 3.3. Chili

Chili pepper (*Capsicum*) is also part of Mesoamerican food and is from the milpa components. Mendivil et al. have previously reported on the anti-inflammatory and anti-fibrotic effects of capsaicin, the bioactive molecule found in chili peppers that is responsible for their pungent taste (Table 1). This was demonstrated in an experimental model of gastritis and liver fibrosis [32]. Additionally, they investigated the protective effects of capsaicin in combination with sulforaphane, a bioactive molecule primarily found in broccoli sprouts, in an experimental model of liver fibrosis [33]. Capsaicin also has antioxidant and anti-steatosis effects [34] and is a highly selective agonist of transient receptor potential vanilloid 1 (TRPV1) channel, a mechanism through which this molecule participates in the amelioration of metabolic disorders [35,36,37]; TRPV1 activation prevents the development of MAFLD through PPARδ-dependent autophagy [38], and modulation of AMPK, PPARα, UCP1, and CLP-1 [35,36]. Treatment with this pungent agent in animal models has shown increased PPARγ activity in adipose tissue of mice fed a high-fat diet [35,39]. On the other hand, dietary supplementation with capsaicin has been a positive intervention in populations at high risk of developing MAFLD, as it prevents the accumulation of hepatic fat [35,37]. In addition, the activation of AMPK in the liver induced by the effect of capsaicin on the TRPV1 receptor is in turn mediated by the increase in adiponectin concentrations, attenuating hepatic inflammation in response [35]. Shin et al. evaluated the effect of topical application of capsaicin on hepatic lipid accumulation, lipogenesis, and fatty acid oxidation through AMPK activation in a murine model of MAFLD induced by high-fat diet. Among the results observed, the following are described: treatment with topical capsaicin decreased hepatic fat at levels of animals fed a standard diet. The data indicate that the effects of capsaicin are associated with adipose tissue which in turn impacts less fat accumulation in the liver. Capsaicin application promoted CD36 and carnitine palmitoyl transferase expression, related with β-oxidation, and fatty acids influx of liver. In addition, capsaicin increased levels of adiponectin, a hormone whose levels are decreased in patients with obesity and NASH. Adiponectin antagonizes lipid accumulation in the liver and prevents inflammation and fibrosis [35].

On the other hand, the proposed mechanism of action of capsaicin is to enhance thermogenesis and modify the intestinal microbiota, promoting weight control at normal levels. In the intestine, capsaicin stimulates the expression of glucagon-like peptide 1 and increases the presence of beneficial bacterial species such as *Akkermansia muciniphila* [37]. Continuous consumption of chili in animal models has shown decreases in serum levels of glucose, cholesterol, and triglycerides [37,40] and their administration in animal models fed a high-fat diet has decreased the weight of the treated animals by 50% [37]. In human studies, in non-obese subjects, the administration of capsaicin decreased the desire to eat, which favored a decrease in energy consumption due to the satiety effect; in addition, capsicum capsaicin-derived molecules lowered plasma glucose levels [37,41]. In the case of capsaicin administration in obese subjects, the effects observed were a change in post-prandial energy expenditure, which increased [37,42,43].

### 3.4. Maize

The pre-Columbian natives of Mesoamerica domesticated and worshipped some of the principal plant foods, maize first among them [44]. In general, maize has the property to decrease the risk of developing non-communicable diseases [10]. The consumption of corn favors the consumer’s health thanks to its broad spectrum of beneficial effects, among which are antioxidant, antihypertensive, anticancer, anti-inflammatory, anticholesterolemic, antimicrobial, and immunomodulatory [45]. The inclusion of pigmented corn in the diet has been shown to favor the delay in the development of non-communicable diseases associated with oxidative stress development processes [46,47]. However, excessive consumption of ultra-processed foods has been linked to celiac disease [48], and there are several ultra-processed corn-derived products in the food industry that may be harmful for individuals with celiac disease.

Purple/blue corn owes its pigment to the high content of cyanidin violet and peonidin-based anthocyanins (Table 1) associated with their biological activities [46]. Magaña-Cerino et al. evaluated the effect of nixtamalized blue corn on oxidative stress and steatosis in an animal model fed a high-fat diet. The dietary strategy decreased ALT and AST levels, increased antioxidant capacity, and decreased lipid peroxidation, and treated animals showed a significant reduction in liver inflammation, decreasing liver damage [46].

Muñoz-Cano et al. examined the effect of traditional Mexican food on an animal metabolic syndrome model. The diet supplementation included maize tortilla and maize pozol (a Mexican traditional beverage made with maize, cacao, and water); the results showed that both supplementations decreased triglycerides and LDL, also both strategies presented a protective effect against the hepatotoxic effect of the sucrose based on the enzyme ALT levels that were similar to those in the control group [10].

In addition, enzymatic hydrolysis of corn gluten generates peptides as products, which have been suggested as a preventive and corrective treatment for MAFLD [49]. These peptides have been shown to be components derived from food protein with a specific amino acid sequence. Once the protein is degraded, these peptides are released and exhibit greater biological activity than the protein from which they are derived [50]. Among the beneficial health effects, these peptides are antihypertensive, antioxidants, liver protectors, and promote ethanol metabolism, among other effects [49,51]. With the aim of understanding the mechanism of action of these corn peptides, Yao et al. evaluated the effect of these molecules on hyperglycemia, oxidative stress, hyperlipidemia, hepatic steatosis, and insulin resistance in an animal model and the molecular mechanisms behind these effects in an in vitro model with HepG2 cells. Observations in the animal model led them to the following conclusions: corn-derived peptides decrease oxidative stress and endoplasmic reticulum stress, subregulate proteins related to lipid metabolism, decreasing lipid accumulation in the liver and therefore hepatic steatosis, as well as suppressing liver damage by decreasing ALT and AST. In the in vitro model, they observed that the peptides activate the AMPKα/Sirt1 [49]. In this context, Wei et al. investigated the effect of maize peptides on the regulation of NF-κB/AMPK signaling activation in Kupper cells induced by lipopolysaccharide. Among the results obtained, the following are listed: decreased serum levels of ALT, AST, and inflammatory cytokines in treated animals, as well as inhibition of TLR4 receptor expression and upregulation of JNK, ERK, and p38 in all their phosphorylated forms, and inhibition of NF-κB/AMPK signaling pathway activation in Kupffer cells [50].

### 3.5. Black Beans

Black bean belongs to the legume family. This seed is widely consumed in Latin America. In Mesoamerica, beans are one of legumes that have lost their place as a source of protein in diets. Their components include 17–23% of protein, 15% dietary fiber, 60% starch where 3–5 g of resistant starch per 100 g remains after the cooking process, and polyphenols/flavonoids [52]. Among the polyphenols of black bean are the anthocyanins (Table 1), to which part of the beneficial effect of this legume is attributed. Anthocyanins are molecules contained in natural red, violet, and blue pigments, and are commonly found bound to sugar molecules. These molecules play an important role in the prevention of obesity and diabetes. It is suggested that once absorbed, they may positively modulate GLUT4 in adipose tissue and skeletal muscle and/or affect the gut microbiota and thus impact consumer health [52,53,54]. Evidence shows that their consumption generates other beneficial health effects [52,53]. Hernandez-Velazquez and colleagues evaluated the effects of the consumption of whole cooked bean flour and a bean protein concentrate on body composition, energy expenditure, and glucose metabolism in an animal model fed with a high-fat/high sucrose diet. The results showed that both interventions reduced weight gain and body fat associated with a significant with decreased expression of lipogenic genes in the liver, suggesting that the protein and bioactive molecules including phenolic and flavonoid compounds are suitable as a diet strategy for subjects with type 2 diabetes or with obesity [52]. In addition, Sánchez-Tapia and colleagues evaluated the effect of black beans on the gut microbiota, body composition, energy expenditure, occluding and insulin signaling, among others, in a rat model with high-fat/high sucrose diet. The results showed that black bean consumption reduced body fat, decreased glucose, and insulin levels, serum leptin, lipopolysaccharides, and increased energy expenditure [55]. The author suggests that these results could be mediated by gut microbiota, by increasing bacteria in the *Clostridia* class, like *R. bromii*, *C. eutactus*, *R. flavefaciens*, *R. callidus*, and *B. pullicaecorum*. In conclusion, black beans could prevent insulin resistance and metabolic endotoxemia by modifying the gut microbiota and protect against obesity [55]. In addition, López-Reyes and colleagues suggest that methanolic black bean showed anti-fibrotic effects in a carbon tetrachloride liver injury murine model [56].

One of the non-communicable diseases related to obesity processes is diabetes mellitus. Deminán-Medina and colleagues investigated black bean multigenic effects and regulatory mechanisms in fat tissue related to improvement in subjects with type 2 diabetes mellitus. The methodology chosen by the research group was a murine diabetic model in which the effect of a black bean extract rich in anthocyanins was tested for 5 weeks. Among the results observed in the treated animals, the following stand out: the sequencing analysis of coding and non-coding RNA showed differences in the expression of 406 genes, among which 33 correspond to miRNA, 39 IncRNA, and 3 snRNA, which participate in the regulation of PI3K signaling, NIN/NF-kB, insulin secretion, and the organization of the endoplasmic reticulum. On the other hand, it was observed that black bean extract metabolites directly interact with transcriptional factors such as GATA2 and POU2AF1 or signaling proteins such as AKT, PI3K, PKB, which may control transcriptional activity and impact the process of adipogenesis and finally to function as a protective anti-diabetic agent [53]. In the same context, Sun et al. studied the regulatory effect of anthocyanins from a black bean skin extract on serum metabolites and gut microbiota in a murine model of type 2 diabetes. The treated animals showed significant decreases in serum levels of glucose, insulin and insulin resistance, parameters affected in the pathological process of diabetes. They also observed a decrease in the levels of proinflammatory cytokines such as IL-6, TNF-α, IL-1β, and an increase in the concentration of molecules involved in the control of oxidative stress such as CAT and SOD. The microbiota of the treated animals was modified, where at the genus level, an increase in the abundance of *Adlercreutzia*, *Phascolarctobacterium*, *Bacteroides*, *Akkermansia* among others and a decrease in *Allobaculum*, *Clostridium*, and *Bifidobacterium* were observed, compared to the diabetes control group. *Akkermansia* has a regulatory effect on glucose metabolism and exerts anti-inflammatory effects by inhibiting TNF-α expression. Other genera such as *Adlercreutzia*, *Phascolarctobacterium*, *Bacteroides* participate in the regulation of lipid metabolism [57,58].

### 3.6. Sweet Potato

Sweet potato (*Ipomoea batata* L.; Lam.) belongs to the Convolvulaceae family, its hepatoprotective, anticancer, antidiabetic, anti-inflammatory, antidiabetic, antitumor, antimicrobial, antiobesity, and other activities have been described [59,60,61,62,63,64]. Among the nutritional components of the sweet potato tuber are dietary fiber, proteins, starch, iron, potassium, manganese, copper, B complex vitamins, vitamin E, vitamin C; as well as other bioactive compounds such as anthocyanins, phenolic acids, carotenoids, coumarins, and flavonoids (including myricetin, quercetin, kaempferol, apigenin, and luteolin) (Table 1) [59,64,65]. There are different varieties whose characteristic is the color of their skin; the varieties with light colors have high concentrations of phenolic compounds (i.e., hydroxycinnamic acids), while more intense yellow colors are related to the content of β-carotene, and finally the purple ones are rich in anthocyanins with anti-inflammatory and antioxidant effects [59,63,65].

Some of the effects observed in the liver are described below. Such a hepatoprotective effect has been observed in murine models of liver damage induced by different chemical agents [63]. Administration of sweet potato extracts and/or anthocyanins in murine models decreases levels of liver damage markers such as ALT and AST [66,67,68], as well as serum TG, LDL, and total cholesterol lactate dehydrogenase [69,70]. Its antifibrotic effect was described by Zhang et al., where oral administration decreased hepatic fibrotic tissue in mice intoxicated with carbon tetrachloride [71]. In humans, consumption of sweet potato beverages decreases serum gamma-glutamyl transferase levels in healthy men with borderline hepatitis, one of the liver enzymes [60]. Yang et al. in 2022 reported results of the longitudinal association (follow-up from 2013 to 2019) between sweet potato intake and the risk of developing MAFLD in the adult general population. The results showed that intake of this tuber is inversely proportional to the risk of MAFLD in males [64]. The authors suggest that such protection is due to mechanisms previously described by other authors such as that sweet potato intake improves glucose and lipid metabolism, which decreases liver damage generated by oxidative stress and inflammation [72,73]. However, more studies are needed to know the exact mechanism by which sweet potato decreases the risk of MAFLD.

On the other hand, the most abundant soluble protein in this tuber is sporamin, representing 60 to 80 percent of the total protein. Among the activities described for this protein are dehydroascorbate reductase and monodehydroascorbate reductase, leading to a reduction in free radicals [61,74]. Based on the above, Zhi-dong et al. evaluated the effect of this protein isolated from sweet potato on the differentiation and proliferation of the preadipocyte cell line 3T3-L1. The treatment significantly inhibited adipocyte differentiation and decreased lipid droplet accumulation and cell proliferation [61]. In this context, Kim and colleagues evaluated the anti-obesity effect of carotenoids and anthocyanins extracted from sweet potato in vitro (3T3-L1 cell line) and in vivo in a murine model of obesity. The treatment significantly inhibited lipid accumulation in vitro, and the extracts decreased TG and PPARγ expression. In the in vivo model, oral treatment of the extracts lowered the weight of the treated animals, decreased TG, and histological evaluation showed a protective effect in the liver versus adipogenesis [59]. Fermentation processes affect the amount of bioactive substances in foods. Lee and colleagues administered fermented sweet potato extract to mice with high-fat, diet-induced obesity. The treatment prevented body weight gain and abnormal white fat tissue expansion. An in vitro assay in 3T3-L1 cells treated with the extract showed increased expression of lipolysis-related mitochondrial genes. The browning effects were increased by fermentation with *Lactobacillus* [75]. Similar results were observed by Kang et al., who administered a sweet potato product fermented with *Lactobacillus rhamnosus* to 3T3-L1 cells and mice fed a high-fat diet. The fermented product reduced lipid and TG accumulation, as well as adipogenesis-related genes such as C/EBPα, PPARγ) in vitro. Dietary administration in animals reduced body weight, amount of body fat, adipocyte cell size, as well as serum parameters such as LDL and cholesterol [76].

Shih et al. administered white sweet potato as a meal replacement (daily consumption of 132 g) in 58 overweight workers in a controlled clinical trial, whose controls were workers with a normal diet, for 8 weeks. The parameters evaluated were body weight, body fat, BMI, and anthropometric measurements. The treated group showed a decrease in fat, weight, BMI, and mid-arm circumference, as well as in glycated hemoglobin levels, suggesting that the consumption of this tubercle facilitates weight loss [77].

Table 2 below shows a compilation of preclinical and clinical trials using cocoa, nopal, chili, corn, black bean, or sweet potato supplementation as treatment in pathological conditions related to liver and fat tissue metabolism.

**Figure 2:**
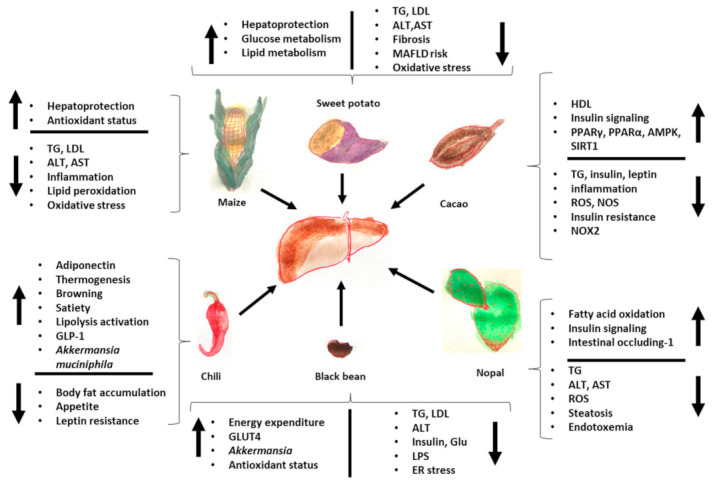
Effects of foods of Mesoamerican origin on liver function. The consumption of nopal, cacao, black beans, chili, and corn has an impact on the metabolism of the liver, improving its metabolic status both in the prevention and treatment of various diseases related to the loss of homeostasis in adipose tissue and in the liver itself. The image shows some of the parameters that increase and decrease with the consumption of each of these foods of Mesoamerican origin. ER: endoplasmic reticulum, TG: triglycerides, GLP-1: glucagon-like peptide, LPS: lipopolysaccharides, ROS: reactive oxygen species, LDL: low-density lipoprotein, HDL: high-density lipoprotein.

**Figure 3:**
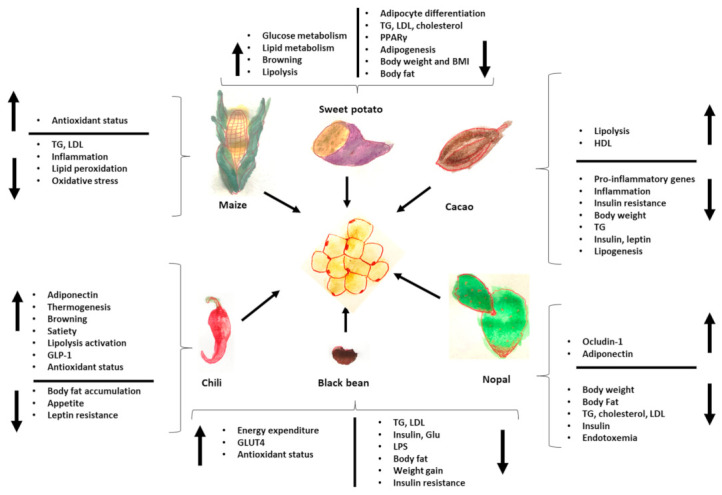
Effects of foods of Mesoamerican origin on adipose tissue. The image shows some of the parameters that increase or decrease in adipose tissue with the consumption of each of these foods of Mesoamerican origin. TG: triglycerides, LPS: lipopolysaccharides, GLP-1: glucagon-like peptide, LDL: low density-lipoprotein, HDL: high-density lipoprotein.

Finally, Figure 2 summarizes the effects of Mesoamerican origin food on liver metabolism and Figure 3 on adipose tissue metabolism.

## 4. Conclusions

Diseases related to the loss of homeostasis of adipose tissue and liver metabolism are on the rise around the world and represent high expenses for healthcare entities in most of the countries on our planet. Mesoamerican origin foods are rich in bioactive molecules that enhance liver and adipose tissue performance and metabolism, suggested in pre-clinical and some clinical results. The pure active molecules, present in the foods described, have been shown to have beneficial effects related to their antioxidant, anti-inflammatory, anti-fibrotic capacity, among others; nevertheless, more scientific research is needed to strengthen the consumption of this type of food so that its consumption increases in society and promotes the prevention of non-transmissible diseases.

## Figures and Tables

**Table 1:** Chemical structures of principal bioactive molecules in foods from Mesoamerican origin.

Bioactive Molecules Family	Compound Name	Mesoamerican Food	Chemical Structure
Flavanols	(−)-epicatechin C_15_H_14_O_6_ 290.27 g/mol	Cacao bean	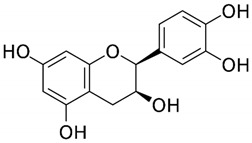
	(+)-catechin C_15_H_14_O_6_ 290.27 g/mol		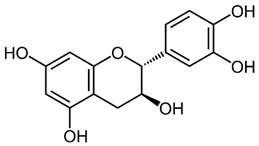
	Procyanidin B2 C_30_H_26_O_12_ 578.5 g/mol		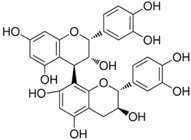
Alkaloids	Theobromine C_7_H_8_N_4_O_2_ 180.16 g/mol	Cacao bean	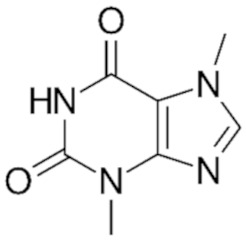
	Capsaicin C_18_H_27_NO_3_ 305.40 g/mol	Chili	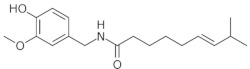
Flavonoids	Quercetin C_15_H_10_O_7_ 302.23 g/mol	Nopal, sweet potato	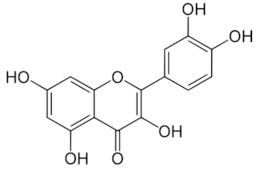
	Myricetin C_15_H_10_O_8_ 318.23 g/mol	Sweet potato	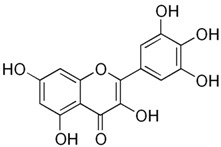
	Luteolin C_15_H_10_O_6_ 286.24 g/mol		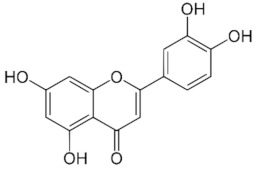
	Apigenin C_15_H_10_O_5_ 270.24 g/mol		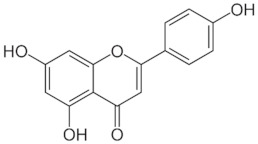
	Kaemferol C_15_H_10_O_6_ 286.24 g/mol		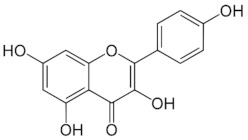
Anthocyanins	Cyanidin C_15_H_11_O_6_^+^ 287.24 g/mol	Blue maize	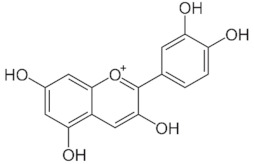
	Peonidin C_16_H_13_O_6_^+^ 301.27 g/mol		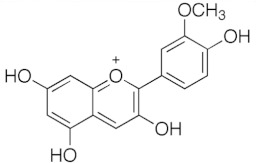
	Delphinidin 3-glucoside C_21_H_21_O_12_^+^ 465.40 g/mol	Black bean	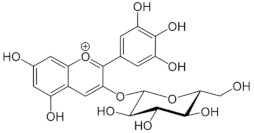
	Petunidin 3-glucoside C_22_H_23_O_12_^+^ 479.40 g/mol		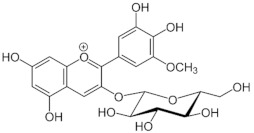
	Malvidin 3-glucoside C_23_H_25_ClO_12_ 528.90 g/mol		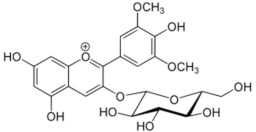

**Table 2:** Compilation of preclinical and clinical studies with foods of Mesoamerican origin for the treatment of conditions related to liver and fat metabolism.

Type of the Study	Food	Intervention	Principal Findings	Author
Pre-clinical	Cacao bean	A total of 8% of Cocoa powder supplementation in HFD-obese mice.	Decrease of inflammation, insulin resistance, and lipid accumulation in the liver.	Gu et al. [26]
		In vitro administration of the alkaloid theobromine, a cocoa component.	Regulation of the lipolysis process and fat oxidation.	Jang et al. [27]
		Administration of cocoa-derived proteins in a murine model of obesity	Overregulation of PPARγ, PPARα, AMPK, Plin1, SIRT1, and PGC-1alpha; and downregulation of TNFα, Leptin, ACC, and SREBP-1c. Genes related to white adipose tissue dysfunction in obesity.	Coronado-Cáceres et al. [29]
Pre-clinical	Nopal	Dietary intervention with a mixture of Nopal, Cacao, and cricket powder in obese HFD-fed mice.	Supplementation decreased body weight, amount of fat, serum parameters such as TG, cholesterol, and LDL, and other hormonal parameters such as insulin, leptin, and resistin. Supplementation increased the amount of beneficial intestinal bacteria.	Rosas et al. [23]
		Oral supplementation of 4% nopal in obese Zucker rats.	The inclusion of nopal in the diet of obese rats decreased the accumulation of hepatic TG, as well as markers of liver damage such as ALT and AST, ROS, and a lower degree of lipid peroxidation. Nopal consumption increased fatty acid oxidation, decreased oxidative stress, and improved insulin signaling.	Morán-Ramos et al. [31]
		Oral supplementation of 5% nopal in obese HFD-rats.	The consumption of nopal modified the composition of the intestinal microbiota, decreased the endotoxemia process, reduced oxidative stress in adipose tissue, and the accumulation of lipids in hepatocytes.	Sánchez-Tapia et al. [7]
Pre-clinical	Chili	Administration of capsaicin in a murine animal model of liver fibrosis.	Orogastric supplementation of capsaicin showed a hepatoprotective effect.	Mendivil et al. [33]
Pre-clinical	Maize	Addition of blue nixtamalized corn in a murine model with high fat diet.	The experimental diet decreased markers of liver damage, ALT and AST, increased antioxidant status, decreased levels of liver tissue inflammation, and consequent damage.	Magaña-Cerino [46]
		Inclusion of maize products, pozol, and tortilla, in a murine model of metabolic syndrome.	The experimental strategy decreased TG and LDL levels and generated a hepatoprotective effect.	Muñoz-Cano et al. [10]
		Administration of corn-derived peptides in an in vivo murine model.	The main results include decreased oxidative stress and endoplasmic reticulum stress, decreased hepatic lipid accumulation, and protection against hepatic steatosis.	Yao et al. [49]
		Administration of corn-derived peptides to a murine model of liver damage.	Treatment decreased inflammatory cytokines, liver damage enzymes (AST and ALT), TLR4 receptor downregulation, and inhibition of the NF-κB/AMPK signaling pathway in recurrent liver macrophages (Kupffer cells).	Wei et al. [50]
Pre-clinical	Black bean	Dietary strategy of bean meal and bean protein concentrate in murine model of obesity fed with HFD and high sucrose.	The treatment prevented weight gain and reduced the amount of body fat.	Hernandez-Velazquez et al. [52]
		Inclusion of black beans in the diet of obese rats fed a high-fat, high-sucrose diet.	The treated group decreased serum glucose and insulin levels, as well as endotoxemia generated by lipopolysaccharides, and increased energy expenditure.	Sánchez-Tapia et al. [55]
		Administration of anthocyanin-rich black bean extract in diabetic murine model.	The administration of the extract impacts the adipogenesis process on several molecules related to this process. The extract was a protective agent against the development of diabetes.	Damián-Medina et al. [53]
		Supplementation with black bean skin extract in murine model of type 2 diabetes.	The treatment decreased glucose and insulin levels. The extract showed anti-inflammatory effect decreasing proinflammatory cytokines, favoring the control of oxidative stress. The supplementation modified the intestinal microbiota, increasing the number of beneficial bacteria.	Sun et al. [57,58]
Clinical	Sweet potato	Association between the risk of developing MAFLD and sweet potato intake in adult population (follow-up from 2013 to 2019).	Regular consumption of sweet potato is inversely proportional to the development of MAFLD.	Yang et al. [64]
		As a meal replacement, 132 g of sweet potato per day was included in the diet of overweight workers for 8 weeks.	The treatment decreased parameters associated with overweight such as body weight, BMI, body fat, among others.	Shih et al. [77]
Pre-clinical		Treatment of 3T3-L1 cells with sporamin (soluble sweet potato protein).	Treatment inhibited preadipocyte differentiation and intracellular lipid accumulation.	Zhi-dong et al. [61]
		Oral administration of carotenoid- and anthocyanin-rich extract of sweet potato in a murine model of obesity.	Supplementation decreased body weight, TG levels, and had a hepatoprotective effect.	Kim et al. [59]
		Supplementation with fermented sweet potato extract in a murine model of obesity.	Treated animals showed no abnormal expansion of white fat tissue.	Lee et al. [75]
		Administration of sweet potato extract fermented with *Lactobacillus rhamnosus* to animals fed a high-fat diet.	The treatment decreased parameters”rela’ed to adipogenesis, such as weight gain, corporal amount of fat, adipocyte size, etc.	Kang et al. [76]

HFD: high-fat diet.

## Data Availability

The data used to support this study are included within the article as references.
